# P-602. Examining the Performance of Novel Vaccines in Large Vaccine Efficacy Trials (1995-2019)

**DOI:** 10.1093/ofid/ofae631.800

**Published:** 2025-01-29

**Authors:** Lauren Jatt, Lawrence Corey, Peter B Gilbert

**Affiliations:** University of Washington, Seattle, Washington; Fred Hutchinson Cancer Research Center, Seattle, WA; Fred Hutchinson Cancer Center, Seattle, Washington

## Abstract

**Background:**

Vaccines are widely recognized as one of the most effective health interventions in the world. The development of novel vaccines against the world’s deadliest diseases is a global health priority. Large, randomized clinical trials are the main mechanism used to determine the efficacy of a vaccine prior to licensure. These trials are expensive and resource intensive. Given the public health importance of these trials, understanding how often they succeed vs. fail and which pathogens they are targeting is paramount to global vaccine programs.

Vaccine efficacy estimates for large vaccine clinical trials against novel pathogens

**Figure:**
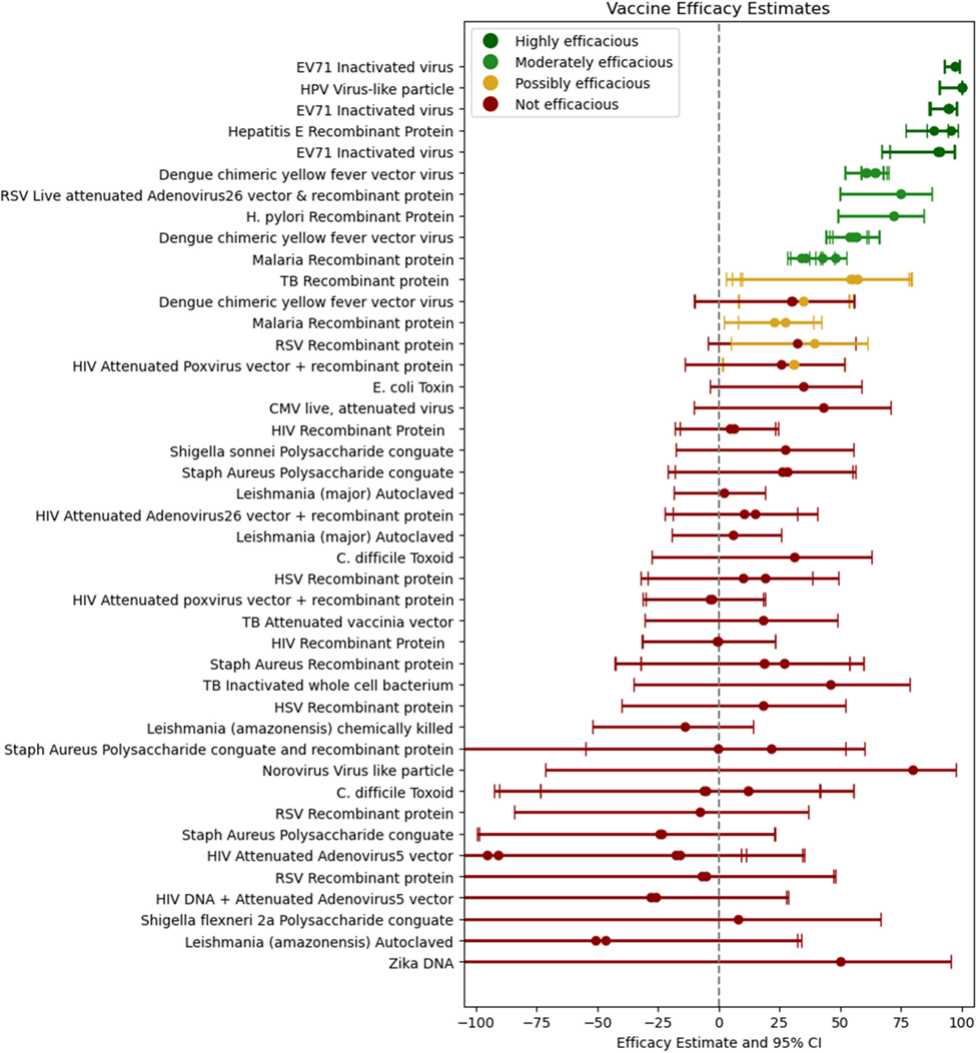

Vaccine efficacy Definitions: highly efficacious (lower bound vaccine efficacy 95% confidence interval ≥65%), moderately efficacious (lower bound vaccine efficacy 95% confidence interval 10-65%), possibly efficacious: lower bound of any of the 95% confidence intervals were 0-10%, and not efficacious: lower bound of any of the 95% confidence intervals were <0.

Abbreviations: Enterovirus 71 (EV71), Human Papilloma Virus (HPV), Respiratory Syncytial Virus (RSV), Cytomegalovirus (CMV), Human Immunodeficiency Virus (HIV), and Herpes Simplex Virus (HSV).

**Methods:**

We searched Pubmed and the International Clinical Trials Registry Platform and identified large (≥ 1000 participants), randomized, vaccine efficacy trials completed between 1995-2019 against infectious disease pathogens for which no efficacious vaccine previously existed.

Summary of characteristics of large vaccine efficacy trials: Distribution by journal of publication, funding source, and pathogen

**Figure:**
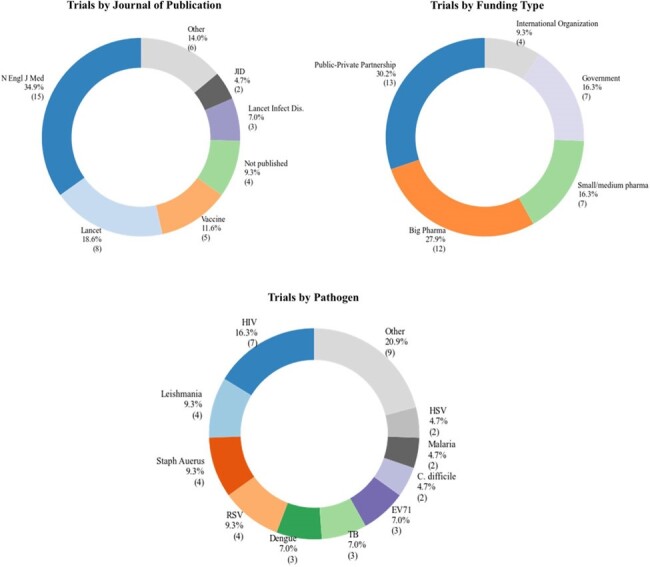

**Results:**

Using the search criteria above, 3483 unique trials were identified in the International Clinical Trials Registry Platform (ICTRP) and 1867 unique citations were identified through Pubmed for a total of 5350 unique entries. During the study period, 43 trials were identified which evaluated vaccines against 18 novel pathogens. Highly efficacious vaccines were developed against EV71, Hepatitis E, and HPV (Figure 1). Moderately efficacious vaccines were developed against Dengue, H. pylori, Malaria, and RSV. A possibly efficacious vaccine was developed against Tuberculosis. Vaccine candidates against ten other novel pathogens failed: C. difficile, CMV, E. coli, HIV, HSV, Leishmania, Norovirus, Shigella, Staph aureus, and Zika. We summarized trial characteristics by journal of publication, funding type, and pathogen (Figure 2). We also performed an exploratory analysis to investigate which studies might have been underpowered and found that the trials for vaccine candidates against Norovirus and Zikavirus were possibly underpowered (Figure 3).

Exploratory sub-analysis conducted to identify trials that were possibly underpowered

**Figure:**
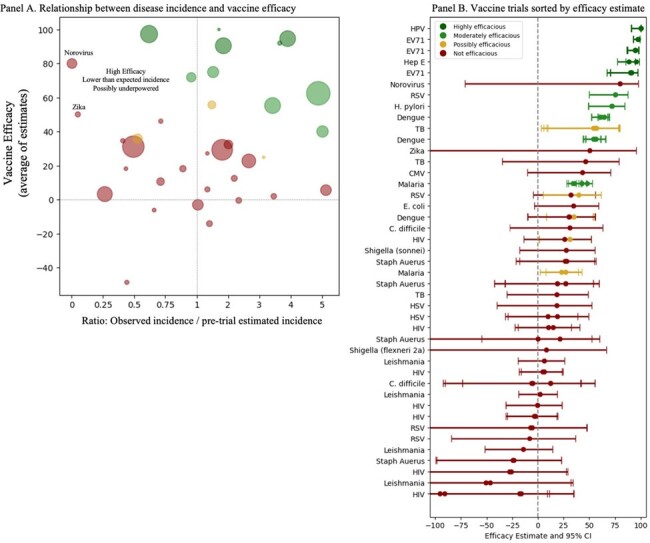

Panel A: Trials in the upper left quadrant represent trials with high efficacy but a significantly lower than expected incidence (compared to the pre-trial estimated incidence that was used to determine the sample size needed to have sufficient power to prove efficacy). Panel B: Vaccine trials are sorted by efficacy estimate, demonstrating that there were some trials that had a high efficacy estimate but wide confidence intervals which might indicate underpowering.

**Conclusion:**

The past quarter century has seen both major successes in new vaccine development and failures. The HPV vaccine has been deployed around the world and is expected to save millions of lives. The malaria vaccine became the first effective vaccine licensed against a parasite. However, many promising candidates failed. Continued efforts to predict efficacy prior to large efficacy trials are needed.

**Disclosures:**

**Lawrence Corey, MD**, NA: HSV Antigens|The Vaccine Company: Ownership Interest|Vir Biotechnology: Ownership Interest **Peter B. Gilbert, PhD**, Sanofi: Advisor/Consultant|Sanofi: Dengue Vaccine research contracts

